# Elevation through reflection: closing the circle to improve librarianship

**DOI:** 10.5195/jmla.2020.938

**Published:** 2020-07-01

**Authors:** Jolene M. Miller, Stephanie Friree Ford, Anna Yang

**Affiliations:** 1 jolene.miller@utoledo.edu, Director, Mulford Health Science Library, and Assistant Professor, Library Administration, University of Toledo, Toledo, OH; 2 sfrireeford@partners.org, Manager, Library Resources, Mental Health Sciences Library, McLean Hospital, Belmont, MA; 3 ayang89@mail.fresnostate.edu, Science Librarian, University Library, California Health Sciences University, Clovis, CA

## Abstract

Reflective practice is a strategy promoted as a way to improve professional performance and to develop expertise. Intentional reflection on work situations can lead to improved understanding of a specific situation, identify strategies for similar situations in the future, and uncover assumptions that hinder service to patrons. Research has identified lack of knowledge to be a barrier to health sciences librarians engaging in reflective practice. This article introduces the use of intentional reflection at work: what it is, how it helps, and how it can be applied in librarianship. It also provides practical advice on how to choose a format, how to use a model to guide reflection, and how to incorporate it into work.

**Figure:**
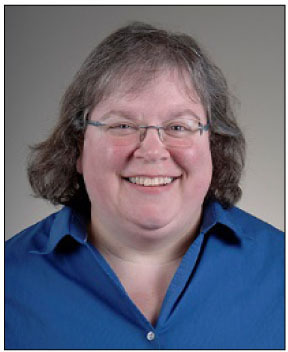
Jolene M. Miller, MLS, AHIP

## INTRODUCTION

In her 2017 article in the *Journal of Information Literacy*, Corrall captures the challenge of incorporating intentional reflection into professional practice: “reflection is a deceptively simple idea that is easy to grasp at a basic level but may be hard to put into practice in a professional [librarian] context” [1]. It is an everyday process that often happens without conscious thought, but reflection with the goal of improving professional practice requires intention. Reflective practice is the process of bringing intentional reflection to one's work to improve practice: providing better instruction, managing electronic resources more effectively, interacting with coworkers more collaboratively, and so on. It closes the loop: new understandings are applied to personal and organizational processes to improve performance. Discoveries about oneself have an impact on thoughts, feelings, and behavior; and relationships improve.

The good news is that no one is a blank slate with respect to reflection at work. The language of reflective practice and the use of models may be new, but the experience of thinking back on a situation and trying to make sense of it is universal. Having a formal process of reflective practice can help health sciences and medical librarians identify and develop best practices. This article, born out of an immersion session offered at the Medical Library Association (MLA) 2019 annual meeting [2], is designed to help readers incorporate reflection into their professional practice.

There are many diverse published models of reflective practice (supplemental Appendix A). They all have three main components: (1) description of the experience, (2) reflection on and exploration of why things happened as they did, and (3) identification of changes to thinking and behavior to improve the outcomes of future experiences. Reflective practice usually starts with consideration of a specific experience that had an unexpected outcome. Most models include guiding questions to make sure that all aspects of the experience are considered, such as external aspects (what happened, who was involved) and internal aspects (how one was feeling before, during, and after the experience). External sources of information such as observations of other people or data from evaluation forms may also be included.

After describing the experience, one reflects on it. This is the core of reflective practice. “Why” questions are common in this stage, guiding analysis and interpretation of the experience [3]. This stage includes judgments: What went well in the experience? What could have gone better? What was one's role in the outcome? What important aspects still need to be identified and considered? After reflection, there is an invitation to action. What could have been done differently? How might the outcome have been different? What needs to be done to improve practice in the future? This could be a change in how things are done or how other people are treated. It could also be a change in thinking about and approaching situations with a different mindset [4].

In addition to having different stages and questions, models approach reflective practice from different perspectives [5]. While all reflective practice models encourage deeper thought about a situation, critical reflection models encourage exploration of the assumptions underlying situations, which is a key step in critical librarianship. Use of critical reflective practice can “direct and inform action that carries social and ethical implications beyond the technical execution of library work” [6]. It provides a method for identifying personal and professional values, exploring where thoughts and actions diverge from these values [7, 8], and identifying courses of action that are consistent with these values. It uncovers hidden biases that influence decision making and hinder high-quality service. In the context of critical librarianship, it is used to identify opportunities to dismantle oppressive social structures and systems such as white supremacy, patriarchy, and capitalism [9].

## REASONS TO TAKE THE TIME TO ENGAGE IN REFLECTIVE PRACTICE

One of the greatest human attributes is the ability to think about and reflect on actions and experiences, whether an unexpected flash of self-reflection, a well-thought-out journal entry, or somewhere in between. As Taylor states, “Thinking can be a gift and a curse, depending on how we employ it in our daily lives” [10]. Based on one's mindset, an experience can be used for good or for ill. For example, an employee may use a negative evaluation as a reason to place full blame for their poor performance on others, rather than use it as a sign to explore their strengths and weaknesses, structural challenges, and ways to improve their performance.

Making the deliberate choice to engage in reflective practice harnesses the power of thought to improve professional practice. Using intentional reflection at work offers a variety of benefits. While the process directly benefits the librarian doing the reflecting, the resulting changes can extend out to the library, institution, and the profession. Some of the ways that reflective practice improves professional practice are explored below.

### Uncovering inconsistencies in thought and action

There are often inconsistencies between what people say they believe and how they act. Critical reflective practice can be used to examine espoused theories (what one says one believes) and theory-in-use (how one acts) [11, 12]. Identifying inconsistencies is the first step in understanding them and resolving them. Are they true inconsistencies or nuanced distinctions? What next actions are needed? Reflection can also facilitate the application of professional standards and ethics in practice [13].

### Improving regulation of emotions

Reflective practice can improve regulation of emotions. It allows librarians to approach situations more objectively and less reactively by the process of cognitive reappraisal. Cognitive reappraisal is a way of thinking differently about a situation that changes internal emotional experiences and, in turn, external emotional expression [14], with the goal of modifying emotions that hinder effectiveness. Reflective practice provides space to explore situations and new ways of thinking about them, reducing their emotional impact and the emotional impact of future situations.

### Reducing burnout

Health sciences and medical librarians provide an array of intensive services: systematic reviews, liaison support, evidence-based practice or critical appraisal instruction, and in-depth research assistance. Managing and providing these services can increase stress and burnout. As noted above, reflective practice facilitates cognitive appraisal, which in turn reduces the risk of burnout caused by emotional labor and suppression of emotions.

While reflective practice in and of itself cannot solve organizational issues that lead to burnout, it can be used to explore how role ambiguity and overload contributes to personal stress and burnout [15]. The results of this reflection can be used in conversations with supervisors to improve position-related and structural issues. In addition, reflective practice can reduce the chance of burnout resulting from “over-learning” repetitive and routine tasks [16]. Taking time to reflect interrupts the “hamster wheel” of activity, reconnecting daily work to the importance of health sciences librarians' role in patient care, education, and research.

### Maximizing professional development

Library school cannot prepare graduates for every possible future, especially health sciences and medical librarians. They must take an active role in their continuing professional development. Many attend webinars and training, while others obtain their credentials through MLA's Academy of Health Information Professionals. Reflective practice can be used to get the most out of the time and money invested in both continuing education and the development of a portfolio for the academy. Taking time to reflect before and after continuing professional development activities can improve learning and assist in the application of new knowledge and skills. Reflective practice can also be used for big-picture planning for professional development [17]. While some continuing education courses include reflection, many do not, requiring librarians to take a more proactive role. Suggestions on how to apply reflective practice in continuing professional development are provided in Table 1.

**Table 1:** Using reflective practice for professional development

Reflections	Applications
Professional development over the course of your career	What knowledge and skills do you need to learn to be successful in your current position? Your overall career goals? Consider using the *Medical Library Association Competencies for Lifelong Learning and Professional Success* (available on MEDLIB-ED) to guide your reflection. Even more broadly: what are your overall career goals now? Have they changed over time?
Before participating in a professional development activity	What do you want to get out of this activity? Is there something specific that you want to learn or explore? Someone you want to meet? An idea you want to discuss? Review learning objectives and/or meeting programs or abstracts before and during reflection.
After the activity	What did you learn in the activity? If it does not match what you hoped to learn, what follow-up do you need to do to achieve your learning goals? Are there things that you want to do differently in your work? If so, what are they? Did you learn anything that changes how you understand your work and identity as a librarian? What impact might this have on your practice? What do you want to learn next? How will you learn it?
Special considerations for multiday meetings	The schedules of multiday meetings are often dawn-to-dusk meetings, papers, keynotes, networking, and more. Take time to reflect occasionally during the meeting. If this ends up as a simple “brain dump” with little reflection, that is okay. The key is to capture your thoughts and ideas. In-depth reflection can take place after the meeting. Consider what you have learned so far, questions you have, programs you could implement in your library, new research ideas, new people you have met, etc. Given your goals, what are your next steps? After multiday meetings, consider blocking out time on your schedule for reflection. It is very easy to fall back into day-to-day work and forget to take time to reflect.

### Demonstrating professional performance

While reflective practice works best when the librarian wants to be engaged in reflection, some institutions require self-reflection as a part of the performance evaluation process, such as part of a portfolio [18, 19]. Because meaningful reflection requires privacy and a trusted environment, reflection on one's performance takes place before the self-evaluation document is written. The document reports on the outcomes of the self-reflection, rather than the full reflective process.

## FORMATS FOR REFLECTIVE PRACTICE

Reflective practice can take on a variety of formats that can be used with a model, combination of models, or no model. Find the formats that work best, remembering that as needs change, so might preferred formats.

### Reflecting alone without recording the reflections

The simplest way to engage in reflective practice is to reflect alone without recording the reflections. This is somewhat similar to meditation in that it is internal reflection; however, analysis and judgment occur that are absent in meditation. This format is a good option if time for reflective practice is short. It is better to think about a situation and not record, than to skip reflection altogether because of lack of time. One drawback to this format of reflective practice is that it relies on memory to track the outcomes of reflection.

### Reflecting alone and capturing the reflection

Reflecting alone and recording the reflection is a popular format for reflective practice. Reflective writing (handwritten journal entry or typing an electronic document) is a common way to reflect, though audio or video recordings can also be used. The obvious benefit of this format is that the reflection is captured for future review. A less obvious but more important benefit of reflective writing is that the writing “is the reflective process,” rather than just a recording of the reflection [20], because the physical process of writing helps clarify thoughts and conclusions. Librarians who are not comfortable with writing could use a form (perhaps based on steps in a reflective practice model) to jot down key ideas from reflection.

### Talking with another person, such as a colleague or mentor

For some, talking through reflection can be effective. Reflective conversation with another person, such as a colleague or a mentor, can lead to additional insight. An outsider's perspective and questions might shed some light on the situation and increase understanding. These conversations need to take place in an environment that is confidential in order to explore situations fully and honestly. Reflective conversations can also be held with a supervisor, though the power differential may hinder deep reflection and honesty. Reflective conversations have additional aspects that need to be considered, which are listed in supplemental Appendix B.

### Talking with a group of people

Small group discussions are another format for those who would like to verbalize their reflections. Group members can support one another in their reflections and learn from each other's successes and mistakes. It can be a regularly scheduled meeting or one called specifically when someone is looking for outside perspectives. Health sciences and medical librarians who work in different environments (e.g., academic, hospital, corporate) can consider forming groups with the intention of conducting reflection. Having some commonalities and some differences supports rich discussion. While reflecting in a group has similar considerations as reflecting with one other person (confidentiality and potential power differentials), it has additional considerations, such as how the group will be facilitated. Supplemental Appendix B lists considerations for being part of a reflective small group.

### Reflecting alone, followed by talking with others

Another option is a combination of reflecting alone and then talking with others, either one-on-one or in a group. This is particularly helpful for those who have trouble identifying the outside perspective or overcoming other challenges in the reflective process. Talking to one or more colleagues can foster deeper individual reflection. Personality, preference, and opportunity will have an impact on format choice. Regardless of the chosen formats, reflection can be freeform or follow a model.

## USE OF A MODEL

There is no formal model for conducting reflection that is geared toward health sciences and medical librarians; however, there are many published models in other professions. A reflective practice model can be used exactly as described in the literature or it can be used as a loose guide. As noted above, it can be turned into a form, where brief answers or comments can be jotted down without extra narrative. Models often provide a visual structure of the reflective process, which allow individuals who are reflecting to incorporate a process for thinking about their experiences, rather than have thoughts float around out of context [10]. Using a model can be particularly helpful for people who are new to reflective practice or for those who want to deepen their practice. Three example models are described below. Additional models can be found in supplemental Appendix A.

### Situation, Evidence, Action (SEA)-Change Model

There are varied reflective practices models from many disciplines, yet the nature of intentional reflection requires the three components described earlier: description of the experience, reflection on and exploration of why things happened as they did, and identification of changes to thinking and behavior to improve the outcomes of future experiences. Some models—such as the Situation, Evidence, Action (SEA)-Change Model (Table 2) that originated in professional library education—focus on these three elements. Instead of including generic questions to guide reflection, the SEA-Change Model identifies characteristics of deep reflection, as defined by Moon [21]. While the complete model includes a de-scaffolding component to help instructors facilitate student autonomy during reflection, leading to independence, the core of the model is three steps: identify the situation, provide the evidence, and follow through with an action or change [22].

**Table 2:** Situation, Evidence, Action (SEA)-Change model

SEA-Change process phases		Characteristics of deep reflection process
S: Situation	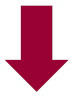	Trigger and/or catalyst: clearly defined and understood. Context: contextual consideration fully considered. Critical (analytical or deep) reflection of multiple perspectives.
E: Evidence	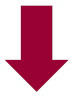	Assimilation of the evidence from the past or present. Learning process based on evidence, new knowledge, or understanding acquired.
A: Action	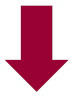	Need for action identified based on above. What action or change is needed? Future Action or change in behavior or the situation
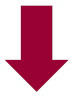 **Change and continued reflection**		

Adapted by permission of Barbara A. Sen.

### Gibbs Reflective Cycle Model

Other models, such as Gibbs' Reflective Cycle Model (Table 3) from the literature of teacher education, include more stages—"Description,” “Feelings,” “Evaluation,” “Analysis,” “Conclusion,” and “Action Plan”—and provide guiding questions to foster a more complete reflection [23]. Even with these extra stages, the three core components of reflective practice are present.

**Table 3:** Gibbs Reflective Cycle Model

Description	Describe your experience.
Feelings	How did you feel during and prior to the experience? How did you react? Describe your feelings after the experience.
Evaluation	What worked and didn't work during the experience? What was the outcome of the experience?
Analysis	Things that went well: Why do you think it went well? How can you improve for the future? Things that didn't go well: Why do you think it didn't go well? What could have been done to avoid this? Think about your contribution to the experience as well as others who were present. Explain if other's reactions were similar or different from yours.
Conclusion	What could you have done differently? What did you learn from this experience, either positive or negative? How did the experience impact your current knowledge?
Action plan	What do you need to do to have a better experience in the future? Even if the experience was positive, what can you improve on? What steps do you need to take to improve for the future?

Used under Creative Commons Attribution NonCommercial 3.0 Unported License by Oxford Centre for Staff and Learning Development, Oxford Brookes University, Wheatley Campus, Wheatley, Oxford, UK, OX33 1HX.

### Kim's Critical Reflective Inquiry Model

**Figure 1:**
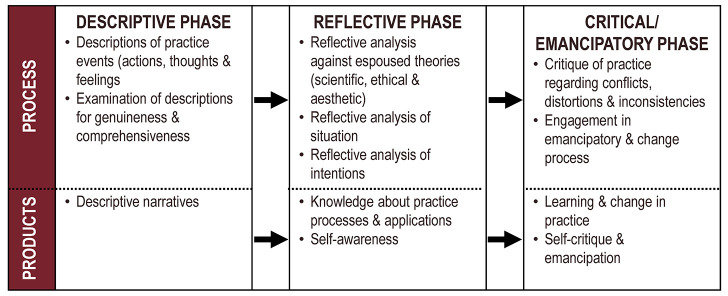
Kim's Critical Reflective Inquiry Model

Some models apply the three main elements of reflective practice to a specific focus. Kim's Critical Reflective Inquiry Model (Figure 1), from the literature of advanced nursing practice, describes three phases: “Descriptive,” “Reflective,” and “Critical/Emancipatory” [24]. In addition to reflection on the situation, it explicitly calls for reflection on one's espoused theories and intentions. This provides appropriate material for the final stage: critiquing practice and participating in the process that leads to change. The key aspect of this model requires critiquing beliefs, assumptions, and personal and professional values. Because of the nature of critical reflective practice, this model benefits from a combination of individual reflection and reflective conversation with others.

Copyright 1999 by John Wiley and Sons. Used with permission.

These three models are a small sample of published reflective practice models. When reviewing models for possible use, consider whether you are:

- New to reflective practice? Consider a model that includes guiding questions.
- Short on time? Consider a simple three-component model.
- Interested in a specific theoretical underpinning? Some models are highly informed by specific theories; others are more practical. Make sure to review the theories used and the assumptions made by potential models. For example, Ghaye offers a strengths-based model [25].
- Interested in creative expression? There are models that incorporate storytelling [26] and poetry [27] as part of the reflective process.

Remember that all models include describing a situation, using reflection to make sense of it, and identifying and making appropriate changes.

## REFLECTIVE PRACTICE IN ACTION

The beauty of reflective practice is that it can be used by health sciences and medical librarians in any type of library, in any type of work, and at any stage in their careers. There are many ways in which reflective practice can be applied to librarianship.

### Evidence-based library and information practice

Reflective practice is an important component of evidence-based library and information practice (EBLIP) [28, 29]. Koufogiannakis and Brettle state that “EBLIP asks librarians to think critically about their practice and the process they use in making decisions” [30]. Reflection is embedded throughout the process, starting with an articulation of the problem to solve or decision to be made and ending with evaluation of the implemented solution or decision made. The journal *Evidence-Based Library and Information Practice* has a column called “Using Evidence in Practice,” which provides a place for librarians to share their experiences with applying evidence to a situation, including a reflection on their processes.

Reflective practice can also be used to improve librarians' skills as creators of evidence. Some authors focus on specific techniques, such as reflective writing [31], while others situate reflective practice in the broader context of research [32, 33]. *Hypothesis*, the journal of the MLA Research Caucus, recently introduced a column called “Failure,” which is an opportunity for health sciences library researchers to reflect on challenges in the research process and how the challenges could have been avoided, allowing others to learn from their experiences [34].

### Critical librarianship

Critical librarianship, “bringing social justice principles to…work in libraries” [35], relies on critical reflection to explore areas where libraries and librarians are supporting systems of oppression and to identify alternatives [36]. Articles on critical librarianship often identify examples and questions from librarianship or other disciplines that can be incorporated into critical reflection. For example, the #CritLib moderators post questions to be discussed at upcoming Twitter chats that can be adopted for individual critical reflection [35], and questions from parts of the chats are archived on the website. Regardless of the source of guidance for critical reflection, it is important to critical librarianship that the reflection results in action: “linking reflection to action is the enactment of critical *practice*” [6].

### Improvement of teaching

Teacher education has a long tradition of reflective practice to improve instruction and classroom management, resulting in a large body of knowledge that has informed the professional development of librarians in teaching roles. Reflective practice has been promoted as a tool for improving teaching skills [37–39] and as a technique for developing one's identity as a teacher [4, 38]. The Association of College & Research Libraries' Framework for Information Literacy for Higher Education has been suggested as a tool to guide reflection with the purpose of improved instruction [40]. Reflective practice is a key component in the development of a teaching portfolio [18].

### Management and leadership

Reflective practice is a helpful tool for administrators, both for helping employees improve their performance and improving the supervisor's own practice. An institution may require employees to complete self-evaluations of their performance prior to the supervisors' evaluations. These annual self-evaluations can be difficult as employees struggle to remember a year's worth of activity. Reflective practice can mitigate the process. Supervisors can encourage their employees to reflect on a regular basis, whether it be monthly, quarterly, or biannually, as an effective way for employees to review their own work and track what was done, constructively gain insight into their performance, and document outcomes of practice improvement processes. This use of reflective practice enables thoughtful consideration of performance and can be used in informal or structured ways.

Reflective coaching can be used with employees between annual evaluations [41]. Reflective practice is beneficial to individual managers and to management teams. Just as the act of reflecting on one's own past work is a helpful tool for employees, it is also helpful for managers. It is a learning process itself, and that learning enables future change and a higher understanding of one's own strengths and weaknesses. Beyond specific work situations, resources are available to use reflection to explore core aspects of leadership [19, 42].

## CONCLUSION

It is not easy to engage in reflective practice. It takes time, dedication, and energy, any or all of which can be in short supply. Investing in one's self and one's career to become a better health sciences or medical librarian is worth the cost. Here are some tips to support exploration of reflective practice.

### Pay attention

Reflective practice includes aspects of mindfulness, as only through paying attention can situations that would benefit from reflection be identified, such as surprising outcomes or uneasy feelings [43]. With a mindful approach, one can explore situations and alternatives in a way in which defensiveness is reduced, improving one's ability to plan and take action.

### Be intentional and purposeful

Start each reflection with an intention to guide the time: why reflect on the identified situation? It is easy to start reflecting on a particular situation and then drift to something else. Granted, the tangent might lead to an important discovery, but an intention can help maintain focus. Sample intentions are how to improve an instruction session with negative evaluations or how to work more efficiently with the information technology (IT) department to streamline access to library resources.

### Use a model to get started

As noted above, using a model can help visualize the process and provide a structure for the reflection. Most reflective practice models include a series of questions that can be used to guide reflection. Pick one that seems likely to work well for the current situation (time available, experience with reflective practice, complexity of the intention, and so on). If it does not work as well as expected, try another.

### Find time

McCorquodale advises: “Value yourself enough to take time to reflect on your practice” [44]. One of the most common factors identified as a barrier to engaging in reflection is lack of time [45, 46]. Everyone leads busy lives, and scheduling time for reflection is the first step to incorporating reflective practice into work life. What is needed: scheduling reflection time regularly or reflecting when a situation arises? If regular reflection is desired, when would be the best time and day to schedule it? Is it scheduled after certain kinds of events, such as after each instruction session? How long might a reflection period last? Block out reflection time on the schedule and defend it. Remember why investing time and effort in reflective practice is worthwhile.

### Find space

Finding a space to reflect is as important as making time. The office is not ideal as there are often too many distractions and interruptions. Whether it is a couch in the library or a table at a coffee shop, find a space to conduct reflection. The space should be a place where one feels confident, protected, free from discrimination, and secure to conduct efficient reflection.

### Find support

There are many different types of support that can be utilized to help to make the reflection process easier. Support from a supervisor can help facilitate the process by helping to find time to reflect. This support will allow the supervisor to see an interest in assessing the quality of the employee's work for professional growth. Supplementing independent reflection with conversation with trusted colleagues (individually or in a group) can provide feedback, clarity, support, and accountability. There are multiple opportunities to find a mentor through professional organizations such as MLA, the Association of Academic Health Sciences Libraries, and regional or state associations.

### Be consistent

Reflective practice is not a skill learned overnight. Like all skills, experience brings improvement both in the reflective process and the application of insights from reflection to professional practice. Consider keeping track of reflections and outcomes in order to reflect on them. Reviewing that history can help identify how reflective practice skills have improved. Reflective practice is not a “one-size fits all” methodology, and there will be some trial and error to find what works best. Additional resources about reflective practice can be found in supplemental Appendix C.

Developing a practice of using intentional reflection does not happen quickly or spontaneously. It requires practitioners to be purposeful and build processes for reflective practice. The investment of time and energy in intentional reflection allows health sciences librarians to learn from their experiences and most importantly, helps them close the circle and apply what they have learned to improve their professional practice.

## Data Availability

There are no data associated with this article.

## SUPPLEMENTAL FILES

**Appendix A:** Models of reflective practice

**Appendix B:** Special considerations for reflective conversations

**Appendix C:** Resources for reflective practice
