# Supplementary material for: Elevation through reflection: closing the circle to improve librarianship
Source: J Med Libr Assoc. 2020 Jul 1;108(3):353–63. doi: 10.5195/jmla.2020.938 (PMC7441907; doi:10.5195/jmla.2020.938)
Supplement: Supplementary file 1 — Appendix A: Models of reflective practice [file jmla-108-3-353-s01.pdf]

JMLA Journal of the Medical Library Association

SC
Supplemental content to
J Med Libr Assoc. Jul;108(3):dx.doi.org/10.5195/jmla.2020.938
www.jmla.mlanet.org
© Miller, Ford, Yang 2020

Elevation through reflection: closing the circle to improve librarianship

Jolene M. Miller, MLS, AHIP; Stephanie Friree Ford, MLIS; Anna Yang, MLIS

APPENDIX A
Models of reflective practice

Atkins and Murphy Model: Atkins S, Murphy K. Reflective practice. Nurs Stand. 1995 Aug 2–8;9(45):31–5.

Critical Incident Analysis: Vachon B, LeBlanc J. Effectiveness of past and current critical incident analysis on reflective learning and practice change. Med Ed. 2011 Sep;45(9):894–904. DOI: http://doi.org/10.1111/j.1365-2923.2011.04042.x.

Driscoll's What? Model: Driscoll J. Chapter 2: Supported reflective learning: the essence of clinical supervision? In: Driscoll J, ed. Practising clinical supervision: a reflective approach for healthcare professionals. 2nd ed. Philadelphia, PA: Elsevier; 2007. p. 27–50.

Experience-Reflection-Action (ERA) Cycle: Jasper M. Beginning reflective practice. Andover, Hampshire, UK: CENGAGE; 2003.

Experiential Learning Cycle: Pfeiffer JW, Ballew AC. The experiential learning cycle: a model. In: Pfeiffer JW, Ballew AC. Using structured experiences in human resource development. San Diego, CA: University Associates; 1988. p. 3–10.

Gibbs Experiential Learning Cycle: Gibbs G. Learning by doing: a guide to teaching and learning methods. Oxford, UK: Oxford Centre for Staff and Learning Development; 1988. (Available from: <https://shop.brookes.ac.uk/product-catalogue/oxford-centre-for-staff-learning-development/books-publications/ebooks/learning-by-doing-a-guide-to-teaching-and-learning-methods-by-graham-gibbs-ebook>. [cited 17 Apr 2020].)

Holistic Reflection Model: Bass J, Fenwick J, Sidebotham M. Development of a model of holistic reflection to facilitate transformative learning in student midwives. Women Birth. 2017 Jun;30(3):227–35. DOI: http://doi.org/10.1016/j.wombi.2017.02.010.

Integrated Reflective Cycle: Bassot B. The reflective practice guide: an interdisciplinary approach to critical reflection. New York, NY: Routledge; 2016.

Kim's Critical Reflective Inquiry Model: Kim HS. Critical reflective inquiry for knowledge development in nursing practice. J Adv Nurs. 1999 May;29(5):1205–12. DOI: http://dx.doi.org/10.1046/j.1365-2648.1999.01005.x.

Model for Structured Reflection: Johns C, ed. Becoming a reflective practitioner. 5th ed. Chichester, West Sussex, UK: Wiley/Blackwell; 2017.

Situation, Evidence, Action (SEA)-Change Model: Sen B, Ford N. Developing reflective practice in LIS education: the SEA-Change model of reflection. Educ Inf. 2009;27(4):181–95. DOI: http://dx.doi.org/10.3233/EFI-2009-0884.
