# Supplementary material for: Elevation through reflection: closing the circle to improve librarianship
Source: J Med Libr Assoc. 2020 Jul 1;108(3):353–63. doi: 10.5195/jmla.2020.938 (PMC7441907; doi:10.5195/jmla.2020.938)
Supplement: Supplementary file 2 — Appendix B: Special considerations for reflective conversations [file jmla-108-3-353-s02.pdf]

JMLA Journal of the Medical Library Association

SC

Supplemental content to
J Med Libr Assoc. Jul;108(3):dx.doi.org/10.5195/jmla.2020.938
www.jmla.mlanet.org
© Miller, Ford, Yang 2020

Elevation through reflection: closing the circle to improve librarianship

Jolene M. Miller, MLS, AHIP; Stephanie Friree Ford, MLIS; Anna Yang, MLIS

APPENDIX B

Special considerations for reflective conversations

As noted in the article, conversation with others (either one other person or a group) can be a helpful tool to reflect more deeply and to help put reflection into action. Reflective conversation involves reflecting aloud with a conversation partner who listens, asks questions, and makes observations. Peers often take turns being the presenter and the listener so that everyone will have an opportunity to reflect. Verbal reflection can help the presenter clarify their thoughts and understanding, with the listener's questions and observations helping to further clarify understanding of the situation, see it from other perspectives, identify preconceived bias, and generate ideas for action.

Some things to consider when identifying a potential conversation partner:

- Can they be trusted to hold the reflective conversation in confidence?
- Are there any power differentials, such as one supervising the other, that could cause concern?
- Do they know enough about each other's work to be able to ask appropriate questions? This can be helpful but is not required.
- Is the listener allowed to offer possible solutions?

It is helpful to have a list of questions as prompts or follow-ups to use. Open-ended questions can be used by the listener to draw out more information from the presenter. Questions such as, "How do you feel it went? What have you tried so far? Was this effective?" and, "What do you recall about your own behavior?" will bring about more description and deeper reflection [1].

Sometimes, reflective conversations are one-and-done. Sometimes they are more formal, longer-term arrangements. When establishing a longer-term arrangement, conversation partners will need to work out logistics:

- Where, when, and how often will you be meeting?
- Which reflective practice model will you be using (if any)?
- Will each person have an opportunity to reflect on a situation at each meeting or will only one person present at each meeting?
- What is expected of the person presenting?
- What is expected of the person listening?

After the conversation, take time to reflect alone and with your conversation partner. What was learned? What actions need to be taken? How well did the reflective conversation process work? Like other forms of reflective practice, reflective conversation is a skill to be developed. What could be done differently to make the next reflective conversation better for the presenter and/or listener?

Reflective practice groups have issues in addition to the logistical issues listed above, such as:

- How will facilitation of the group be handled? Consistently the same person or is it a rotating responsibility? If it rotates, do all group members have adequate facilitation skills to be successful?
- What are the group's ground rules to set expectations and guide behavior?
- What will happen at a standard meeting? Does a member reflect on a particular situation, with the others making observations and asking questions? Does the facilitator (or other group member) bring a more generic situation or issue for the group to discuss?

JMLA Journal of the Medical Library Association

SC

Supplemental content to
J Med Libr Assoc. Jul;108(3):dx.doi.org/10.5195/jmla.2020.938
www.jmla.mlanet.org
© Miller, Ford, Yang 2020

For more information on facilitating and participating in reflective practice groups, start with these chapters:

- Bassot B. Chapter 9: Reflecting in groups. In: Bassot B. The reflective practice guide: an interdisciplinary approach to critical reflection. New York, NY: Routledge; 2016. p. 105–16.
- Jasper M. Chapter 6: Ways of reflecting with others. In: Jasper M. Beginning reflective practice. Andover, Hampshire, UK: CENGAGE; 2003. p. 172–98.
- Reale M. Chapter 8: Reflecting with colleagues. In: Reale M. Becoming a reflective librarian and teacher: strategies for mindful academic practice. Chicago, IL: American Library Association; 2017. p. 81–92.

APPENDIX REFERENCE

1. Wormeli R. Reflective coaching: training for all teachers. AMLE Magazine. 2017 Oct;5(4). (Available from: <https://www.amle.org/BrowsebyTopic/WhatsNew/WNDet/TabId/270/ArtMID/888/ArticleID/860/Cognitive-Coaching-Training-for-All-Teachers.aspx>. [cited 17 Apr 2020].)
