# Supplementary material for: Elevation through reflection: closing the circle to improve librarianship
Source: J Med Libr Assoc. 2020 Jul 1;108(3):353–63. doi: 10.5195/jmla.2020.938 (PMC7441907; doi:10.5195/jmla.2020.938)
Supplement: Supplementary file 3 — Appendix C: Resources for reflective practice [file jmla-108-3-353-s03.pdf]

JMLA Journal of the Medical Library Association

SC
Supplemental content to
J Med Libr Assoc. Jul;108(3):dx.doi.org/10.5195/jmla.2020.938
www.jmla.mlanet.org
© Miller, Ford, Yang 2020

Elevation through reflection: closing the circle to improve librarianship

Jolene M. Miller, MLS, AHIP; Stephanie Friree Ford, MLIS; Anna Yang, MLIS

APPENDIX C
Resources for reflective practice

Bassot B. The reflective practice guide: an interdisciplinary approach to critical reflection. New York, NY: Routledge; 2016.

This title provides a good introduction to reflective practice, including overviews of key theories and models of reflective practice and how to put them into practice. It includes chapters on how to work with emotions and assumptions, group reflective practice, and the importance of feedback.

Bolton G. Reflective practice: writing and professional development. 5th ed. Thousand Oaks, CA: SAGE Publishing; 2018.

One of the classic titles in reflective writing, this book discusses principles, ethics, theories, and contexts of reflective writing. It provides many exercises ("write to learn") to develop comfort with writing (not an insignificant barrier for many people) and use of writing in reflective practice. It explores reflective writing for individuals, peers, and groups.

Ghaye T. Teaching and learning through reflective practice: a practical guide for positive action. New York, NY: Routledge; 2011.

Ghaye approaches reflective practice from the perspective of appreciative inquiry and positive psychology, starting from what is right, rather than what is wrong. Written for teachers and student teachers, the content is appropriate for librarians in all aspects of librarianship. It includes suggestions for reflective conversations and learning journals.

Jasper M. Beginning reflective practice. Andover, Hampshire, UK: Cengage Learning; 2003.

This is an excellent introduction to reflective practice, providing overviews of reflective practice models with examples, suggested reflective activities, and guidelines for reflective practice. Clearly a textbook for students, this title has information that is relevant for practitioners in any profession.

Johns C, ed. Becoming a reflective practitioner. 5th ed. Chichester, West Sussex, UK: Wiley/Blackwell; 2017.

This text explores reflective practice from a nursing perspective. The content is based on Johns's Model for Structured Reflection, with five phases and guiding questions. This title promotes reflective writing as well as more creative expressions such as poetry and visual art. The chapters on the use of reflection in nursing education may be less helpful for librarians.

JMLA Journal of the Medical Library Association

Supplemental content to
J Med Libr Assoc. Jul;108(3):dx.doi.org/10.5195/jmla.2020.938
www.jmla.mlanet.org
© Miller, Ford, Yang 2020

Pellicer LO. Caring enough to lead: how reflective practice leads to moral leadership. Thousand Oaks, CA: Corwin Press; 2008.

Focusing on issues of everyday leadership, this book contains essays on themes in leadership, each with questions to guide personal reflection. It is recommended for individual and group guided reflection, regardless of one's formal role in an organization.

Reale M. Becoming a reflective librarian and teacher: strategies for mindful academic practice. Chicago, IL: American Library Association; 2017.

This book is a down-to-earth discussion of reflective practice in academic librarianship that can be easily applied to other areas of librarianship. This guide provides practical examples of and suggestions for how reflective practice can be incorporated into librarians' work. The emphasis is on independent reflective writing with some information on group reflective practice.

Stevens DD, Cooper JE. Journal keeping: how to use reflective writing for learning, teaching, professional insight, and positive change. Sterling, VA: Stylus Publishing; 2009.

Are you new to journaling and reflective writing? This book situates journal keeping in the contexts of reflective learning and adult development. It provides techniques and case studies to illustrate them. The authors address using journals in the classroom and for professional development. The segments on professional development are more relevant for reflective practice.

Wormeli R. Reflective coaching: training for all teachers. AMLE Magazine. 2017 Oct;5(4). (Available from: <https://www.amle.org/BrowsebyTopic/WhatsNew/WNDet/TabId/270/ArtMID/888/ArticleID/860/Cognitive-Coaching-Training-for-All-Teachers.aspx>. [cited 17 Apr 2020].)

This article and the supplemental document linked from the article provide an overview of reflective coaching. This list of questions for reflective coaches is helpful for supervisors to use with employees and for peers in reflective conversations.
